# Probiotic Supplementation Improves Lipid Metabolism Disorders and Immune Suppression Induced by High-Fat Diets in *Coilia nasus* Liver

**DOI:** 10.3390/biology14040381

**Published:** 2025-04-07

**Authors:** Jun Gao, Qi Mang, Yi Sun, Gangchun Xu

**Affiliations:** 1 Freshwater Fisheries Research Center, Chinese Academy of Fishery Sciences, Wuxi 214081, China; gaojun@ffrc.cn (J.G.); sunyi@ffrc.cn (Y.S.); 2 Wuxi Fisheries College, Nanjing Agriculture University, Wuxi 214081, China; mangq@cafs.ac.cn

**Keywords:** *Coilia nasus*, 4D-DIA proteomics, probiotics supplementation, high-fat diet, lipid metabolism, metabolism disorder

## Abstract

High-fat diets (HFDs) usually trigger disruptions in lipid metabolic processes and immune suppression in fish. As an eco-friendly and potent additive, the inclusion of probiotics in fish diets ameliorates dysregulations in lipid metabolism, mitigates oxidative stress, and reduces inflammatory reactions triggered by HFDs. However, little current research has focused on the improvement of the hazards of HFDs in fish by probiotics. Therefore, we employed 4-dimensional data-independent (4D-DIA) proteomic analysis to investigate the mechanism of the protective impact of probiotics against HFD-induced hepatic injury in *Coilia nasus* between the HFD group and the probiotic supplementation in HFD (PHFD) group. Additionally, lipid accumulation and antioxidant indicators in the liver were also measured via Oil Red O staining and activity detection. Administration of probiotics markedly attenuated the hepatic concentrations of triglycerides (TG), cholesterol (CHO), and low-density lipoprotein cholesterol (LDL-C) in *C. nasus* subjected to HFDs. Furthermore, it significantly upregulated the expression of the differentially expressed proteins (DEPs) implicated in cholesterol metabolism and fatty acid oxidation, while concurrently downregulating the DEPs associated with fatty acid synthesis. Additionally, probiotic supplementation significantly reduced the aspartate aminotransferase (AST), alanine aminotransferase (ALT), and malondialdehyde (MDA) levels induced by HFDs. It also upregulated the activities of catalase (CAT) and superoxide dismutase (SOD). Probiotic supplementation significantly upregulated the DEPs related to antioxidants, while significantly downregulating the DEPs associated with inflammatory responses and autophagy. These findings suggested that probiotics ameliorated HFD-induced hepatic lipid accumulation in *C. nasus* by enhancing cholesterol metabolism and fatty acid oxidation, concomitantly with the suppression of fatty acid synthesis pathways. Additionally, probiotics protected against HFD-induced hepatic injury by enhancing antioxidant defenses and suppressing inflammation in *C. nasus*.

## Simple Summary

High-fat diets (HFDs) often disrupt lipid metabolism and induce immune suppression in fish. Probiotics, which are eco-friendly and potent, have been shown to improve lipid metabolism, mitigate oxidative stress, and reduce inflammation associated with HFDs. In this study, we employed 4-dimensional data-independent (4D-DIA) proteomics to elucidate the mechanisms by which probiotics protect *Coilia nasus* livers from HFD-induced damage. We also measured hepatic lipid accumulation and antioxidant levels between the HFD group and the probiotic supplementation in HFD (PHFD) group. The results showed that probiotic supplementation significantly reduced hepatic concentrations of triglycerides (TG), cholesterol (CHO), and low-density lipoprotein cholesterol (LDL-C) in fish-fed HFDs. Probiotics upregulated proteins involved in cholesterol metabolism and fatty acid oxidation while downregulating those associated with fatty acid synthesis. Additionally, probiotics decreased liver damage markers (aspartate aminotransferase (AST), alanine aminotransferase (ALT), and malondialdehyde (MDA)) and increased antioxidant enzyme activities (catalase (CAT) and superoxide dismutase (SOD)). These findings suggest that probiotics protect fish livers by enhancing lipid metabolism, suppressing fatty acid synthesis, and reducing oxidative stress and inflammation. Probiotics thus represent a valuable dietary additive for improving fish health under high-fat feeding conditions.

## 1. Introduction

Fat serves as a vital energy reservoir, supplying energy for decreasing the demand for proteins [1,2]. Recently, intensive aquaculture has gradually increased the demand for high-fat diets (HFD) to provide additional energy or substitute proteins [3]. Nevertheless, an overabundance of dietary lipids can precipitate a cascade of metabolic issues, including the accumulation of excess body fat and the inception of hepatic steatosis, thereby impeding aquaculture advancement [4,5,6].

A growing number of studies has revealed that HFD precipitated disruptions in lipid metabolic processes, culminating in lipid accumulation within fish. The liver of fish is a crucial organ for lipid metabolism. Lipid metabolism mainly involves synthesis, transportation, and oxidative decomposition, and a balance between these three aspects is necessary to maintain normal lipid metabolism [7]. In *Misgurnus anguillicaudatus*, as the proportion of fish oil in feed formulations increased, the increased mRNA levels of stearoyl-CoA desaturase (*scd*) and sterol regulatory element-binding protein 1 (*srebp1*) promoted lipid synthesis, while the decreased mRNA levels of hormone-sensitive lipase (*hsl*), peroxisome proliferator-activated receptor α (*pparα*), and carnitine palmitoyltransferase 1 (*cpt1*) inhibited lipid oxidation [8]. Short-term (30 days) feeding of HFDs activated fatty acid transport protein genes in the liver of Nile tilapia (*Oreochromis niloticus*), while long-term (60 days) feeding resulted in increased triglyceride synthesis and chylomicron accumulation, accompanied by a decrease in fatty acid biosynthesis [9]. Furthermore, long-term feeding of HFDs also raised TG, CHO, and high-density lipoprotein cholesterol (HDL-C) levels within the serum and liver of black seabream (*Acanthopagrus schlegelii*) [10]. HFD-induced lipid metabolic disorders can cause liver injury. Specifically, such diets may lead to elevated levels of ALT and AST, which are indicative of hepatocyte damage. Moreover, they can disrupt the balance of antioxidant enzymes, such as CAT and SOD, and increase MDA levels, reflecting enhanced lipid peroxidation and oxidative stress in the liver [8,9,10].

Adequate fat intake is crucial for the development of aquatic species; however, an overabundance of fat can trigger lipid peroxidation and oxidative stress [11]. In giant freshwater prawns (*Macrobrachium rosenbergii*), chronic consumption of an HFD resulted in oxidative stress and immune suppression [12]. The dietary administration of an HFD to *Scophthalmus maximus* was observed to markedly elevate hepatic MDA levels, concurrently leading to a reduction in the antioxidant enzymatic activities [13]. This phenomenon also occurred in *Ctenopharyngodon idella* [14], and *Larimichthys crocea* [15]. In farmed fish, oxidative stress induced by long-term feeding of HFDs led to inflammatory responses, mitochondrial dysfunction, and cell apoptosis [13]. Additionally, HFDs can induce metabolic inflammation. HFDs increased liver steatosis in black seabream, stimulating nuclear factor κB (NF-κB) and inflammation [6]. The supplementation of an HFD in Nile tilapia resulted in a substantial elevation of tumor necrosis factor alpha (TNF-α) and interleukin 1 beta (IL-1β) [16]. Additionally, the hepatic and intestinal tissues of black seabream subjected to HFDs exhibited a marked increase in the expression of NF-κB and IL-1β, highlighting the pronounced inflammation elicited in these organs [6].

Recently, the application of probiotics in regulating HFD-induced disruptions in lipid metabolism in mammals has received increasing attention. Probiotics modulate cholesterol metabolism through the suppression of cholesterol synthesis and the enhancement of cholesterol transformation. *Lactobacillus rhamnosus* downregulated the mRNA expression of hepatic 3-hydroxy-3-methylglutaryl-CoA reductase in HFD-fed mice, leading to a subsequent decrease in serum cholesterol levels [17,18]. Probiotics accelerated the hepatic transformation of cholesterol into bile acids by secreting bile salt hydrolase or cholesterol reductase, thereby promoting the entry of cholesterol from the blood into the liver and reducing serum cholesterol levels [19]. Probiotics can regulate triglyceride metabolism by promoting triglyceride breakdown and inhibiting triglyceride synthesis. Probiotics can accelerate triglyceride breakdown by activating the PPAR signaling pathway to promote fatty acid beta-oxidation [20]. Supplementation with probiotics significantly reduces serum triglyceride levels in high-fat diet-fed mice, potentially due to increased hepatic PPAR-δ expression and decreased SREBP-1c expression [21]. In fish, feed additives such as chenodeoxycholic acid [22], tocopherols [13], L-carnitine [23], ginkgo biloba [24], and turmeric [11] are widely used to mitigate HFD-induced disruptions in lipid metabolism and immune suppression. However, research is scarce concerning the supplementation of probiotics to ameliorate lipid metabolic disturbances and immune suppression in fish maintained on HFDs.

As a valuable species of *Coilia*, *C. nasus* is a fleshy, tasty, and highly prized migratory fish, widely distributed in major rivers and offshore waters of East Asia. Currently, due to the lack of efficient feeds, the fat content in feeds for *C. nasus* is relatively high, threatening the growth and health of farmed *C. nasus*. Consequently, the prevalent application of HFDs resulting in excessive lipid accumulation has emerged as a significant challenge in farmed *C. nasus*. Adding probiotics is an effective approach to mitigate lipid metabolic disturbances and immune suppression elicited by HFDs. The current investigation endeavors to elucidate the modulatory mechanisms of probiotics on lipid metabolism and immunity via proteomics; our findings offer novel strategies and methodologies for the aquaculture.

## 2. Materials and Methods

### 2.1. Experimental Fish and Design

Healthy *C. nasus* used in the present study were from Yangzhong, China, with a mean length of 11.02 ± 1.25 cm and a mean weight of 5.62 ± 0.68 g. A total of 1800 fish were randomly separated into six ponds (160 m^3^, 300 fish per pond). After acclimatization for a week, these six ponds were divided into the high-fat diet without probiotics group (HFD) (crude fat: 14.58 ± 0.91) and the high-fat diet with probiotics group (PFHD). Based on our previous reports, 1.0 × 10^8^ CFU/g compound probiotics (*Lactobacillus plantarum*: *Saccharomyces cerevisiae*, 9:1) were added to the high-fat diets [25,26,27]. The *L. plantarum* were cultured in LB media at 28 °C for 24 h. The *S. cerevisiae* were cultured in YPD media at 28 °C for 48 h [25]. The bacterial suspension was sprayed onto the diets using drum mixers. Following a 120-day period, 50 fish were randomly selected from the pond using a seine net. After excluding individuals that were injured or died due to stress, as well as those with abnormal growth indicators, five fish were randomly anesthetized using a 75 mg/L solution of MS-222 from each parallel group (15 per group in total). Subsequently, hepatic tissues were expeditiously harvested, immediately immersed in liquid nitrogen for rapid freezing, and subsequently maintained at −80 °C for subsequent analysis. The crude fat of the HFDs used in this study reached 14.58 ± 0.91%, and the detailed contents of the HFDs are shown in Table S1. The *Guide for the Care and Use of Laboratory Animals* was followed during all experimental procedures, and all experimental protocols were approved by the Chinese Academy of Fishery Sciences’ animal ethics committee (No. YZ2024168).

### 2.2. 4D-DIA Proteomics

Sample preparation and fractionation for mass spectrometry: six liver tissue samples (three per group) were processed by homogenization and subjected to SDT buffer and lysis solution treatment. After sonication and a 15-min boiling step, the supernatant was isolated by centrifugation at 14,000× *g* for 40 min, and protein concentration was determined using a BCA Protein Assay Kit from Bio-Rad, Hercules, CA, USA. Proteins from each sample were extracted into the SDT buffer, and the UA buffer was introduced for ultrafiltration to eliminate impurities such as detergents and low-molecular-weight substances such as DTT. The samples were then treated with 100 μL of iodoacetamide and incubated in the dark for 30 min, followed by two washes with 100 μL of the UA buffer and 100 μL of the 25 mM NH_4_HCO_3_ buffer. Peptide extraction was achieved by enzymatic digestion with 4 μg of trypsin in 40 μL of the 25 mM NH_4_HCO_3_ buffer at 37 °C overnight. The resulting peptides were fractionated into ten portions using a Thermo Scientific™ Pierce™ (Waltham, MA, USA) High pH Reversed-Phase Peptide Fractionation Kit. Each portion was concentrated by vacuum centrifugation and redissolved in 15 μL of 0.1% formic acid. Peptide desalting was carried out using C18 cartridges (Empore™ SPE Cartridges C18, 7 mm bed ID, 3 mL volume, Sigma, Tokyo, Japan), and the samples were redissolved in 40 μL of 0.1% formic acid. Peptide concentration was assessed by absorbance at 280 nm with a NanoDrop2000c spectrophotometer from Thermo.

LC–MS/MS analysis. Employing a data-independent acquisition (DIA) strategy, peptide analysis was performed on a nano liquid chromatography–tandem mass spectrometry (nanoLC–MS/MS) system. Spectrometry data were acquired using ion mobility–mass spectrometry (IM–MS) over a mass range of *m*/*z* 100–1700. Trapped ion mobility spectrometry (TIMS) scans, each lasting 100 ms, were executed across eight distinct windows. For the parallel accumulation–serial fragmentation (PASEF) MS/MS analysis, the collision energy scaled linearly with ion mobility, starting at 20 eV for 1/K0 = 0.6 V/cm^2^ and peaking at 59 eV for 1/K0 = 1.6 V/cm^2^.

Mass spectrometry data analysis. The spectral library generated was interrogated utilizing SpectronautTM version 14.4.200727.47784. The key parameters included the application of dynamic iRT for predicting retention times, the activation of interference correction at the MS2 level, and the execution of cross-run normalization. Analysis of the results was filtered based on a stringent false discovery rate (FDR) threshold set at less than 1%. A *t*-test was performed to analyze differential protein expression between the groups. All statistical analyses were conducted as two-tailed tests, with proteins exhibiting a fold change (FC) greater than 1.5 or less than 0.67 and a *p*-value below 0.05 being classified as significantly differentially expressed. These proteins were then mapped to the KEGG database for functional annotation (http://geneontology.org/, accessed on 16 May 2024). Despite issues with accessing the webpage, which may have been due to the link or network-related problems, the mapping process is essential for understanding protein functions and pathways. Pathway enrichment analysis was conducted using Fisher’s exact test, and the Benjamini–Hochberg procedure was applied to control for multiple comparisons and adjust the *p*-values accordingly. Functional categories and pathways with adjusted *p*-values below 0.05 were considered to be statistically significant.

### 2.3. Detection of Biochemical Indexes of the Liver

Three liver tissues per group stored at −80 °C were weighed accurately. They were homogenized in nine volumes of normal saline. The homogenate was centrifuged at 250 rpm for 10 min. Then, the supernatant (10% tissue homogenate) was taken for further analysis. The assays for CAT (spectrophotometric assays, A007-1-1), SOD (WST-1 method, A001-3-2), ALT (Reitman–Frankel method, C009-2-1), and AST (Reitman–Frankel method, C010-2-1) were conducted utilizing commercial kits procured from Nanjing Jiancheng Bioengineering Institute (Nanjing, China). Additionally, the levels of CHO (single reagent, GPO–PAP method, A111-1-1), LDL-C (two-reagent direct method, A113-1-1), and MDA (TBA method, A003-1-2) were measured with kits from the same supplier. TG (spectrophotometer / microplate reader, BC0625) concentrations were assessed using kits obtained from Beijing Solarbio Science & Technology Co., Ltd. (Beijing, China). All measurements were conducted in strict compliance with the protocols provided by the manufacturers. Each sample was analyzed three times.

### 2.4. Oil Red O Staining

Liver lipid accumulation was quantitatively assessed through Oil Red O staining according to standardized protocols with modifications. For each experimental group (*n* = 3 biologically independent animals), three consecutive 6 μm cryosections were collected from three replicates, yielding 9 technical replicates per group. The sections were fixed in pre-cooled 4% formaldehyde (4 °C, 15 min) prior to Oil Red O staining (0.5% *w/v* in 60% isopropanol; Sigma-Aldrich, O1391). After counterstaining with hematoxylin (5 s) and mounting with glycerol gelatin (Sigma-Aldrich, G7765), all the sections were imaged under identical conditions using an Olympus BX53 microscope equipped with a DP74 camera (Olympus, Tokyo, Japan) [28]. Quantification was performed through triple-blind analysis (operator, analyst, and statistician unaware of group assignments) using ImageJ 1.53 with the following workflow: (1) convert RGB images to 8-bit grayscale; (2) apply a uniform threshold (40–65 intensity range) optimized via pilot analysis; (3) calculate the positive area percentage = (thresholded pixels/total tissue pixels) × 100%; (4) exclude regions with sectioning artifacts (<5% of the total area).

### 2.5. Statistical Analysis

A *t*-test was used for the differential analysis of the data performed using SPSS 25.0. Statistical significance was established at a *p*-value threshold of less than 0.05. Histograms were drawn with Prism GraphPad 10.0. Heatmaps were drawn with Tbtools [29]. Circos plots of KEGG enrichment were drawn with OmicShare (https://www.omicshare.com/tools/, accessed on 6 July 2024) [30].

## 3. Results

### 3.1. Effects of Probiotics on Hepatic Biochemical Indexes of the C. nasus Fed HFDs

**Figure 1:**
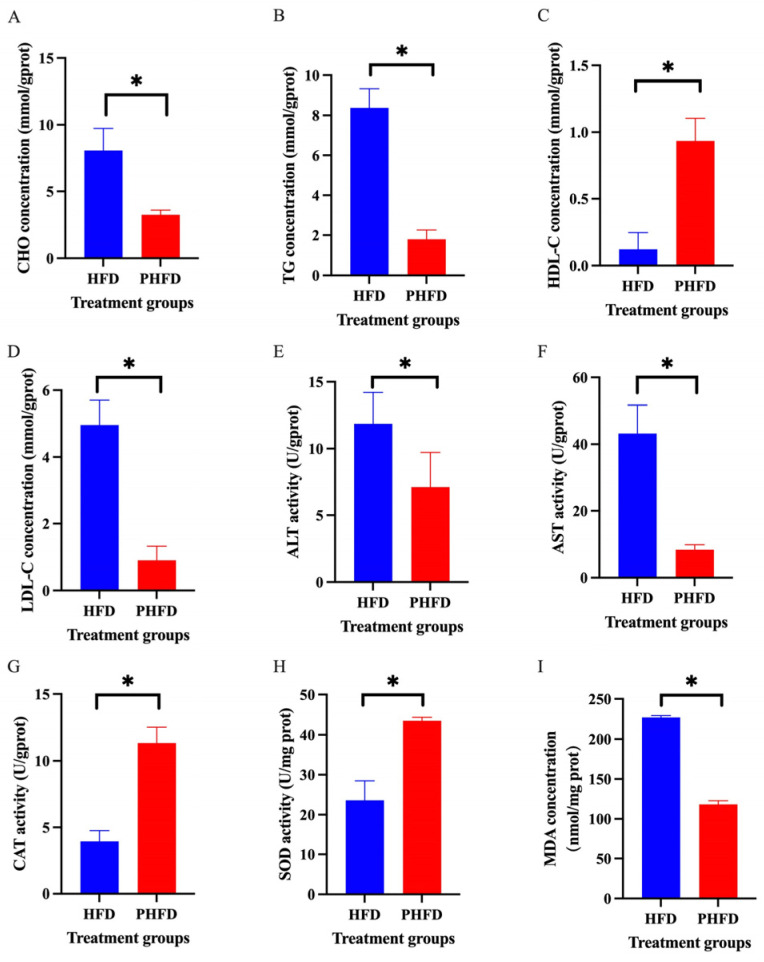
Effects of probiotics on hepatic biochemical indexes of the *C. nasus* fed high-fat diets. Alteration of lipid metabolism indexes, including (**A**) CHO, (**B**) TG, (**C**) HDL-C, (**D**) LDL-C, and hepatic damage indexes, including (**E**) ALT, (**F**) AST, (**G**) CAT, (**H**) SOD, (**I**) MDA between the high-fat diet group (HFD) and the probiotic supplementation group (PHFD). The data were displayed as the means ± SD (*n* = 9). Note: * indicates a significant difference (*p* < 0.05).

The concentrations of CHO, TG, and LDL-C were markedly reduced in the PHFD group (*p* < 0.05) (Figure 1A–C), while the HDL-C concentration was significantly increased (*p* < 0.05) (Figure 1D). The activities of AST and ALT in the PHFD group were inhibited (*p* < 0.05) (Figure 1E,F). The activities of CAT and SOD in the PHFD group were promoted (*p* < 0.05) (Figure 1G,H), while the MDA concentration was diminished in the PHFD group (*p* < 0.05) (Figure 1I).

### 3.2. Effects of Probiotics on Lipid Storage in the Hepatopancreas of the C. nasus Fed HFDs

**Figure 2:**
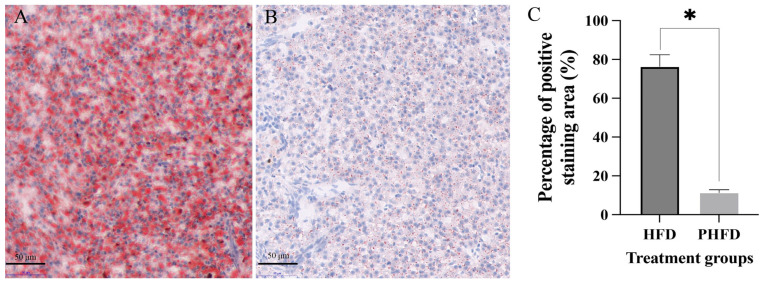
Effects of probiotics on lipid storage in the hepatopancreas of *C. nasus* fed HFDs. Oil Red O staining of the hepatopancreas characterizing lipid accumulation in the HFD group (**A**) and the PHFD group (**B**) (40× magnification). Scale = 50 μm. Quantification analysis of Oil Red O staining (**C**). The data were displayed as the means ± SD (*n* = 45). Note: * indicates a significant difference (*p* < 0.05).

The result of Oil Red O staining of the hepatopancreas in *C. nasus* is shown in Figure 2. The lipid vacuoles in the HFD group (Figure 2A) had a statistically higher number compared to the PHFD group (Figure 2B). Quantification analysis showed that the percentage of the positive staining area in the HFD group was significantly higher than in the PHFD group (Figure 2C).

### 3.3. 4D-DIA Protein Profiling for Differentially Expressed Protein (DEP) Identification

**Figure 3:**
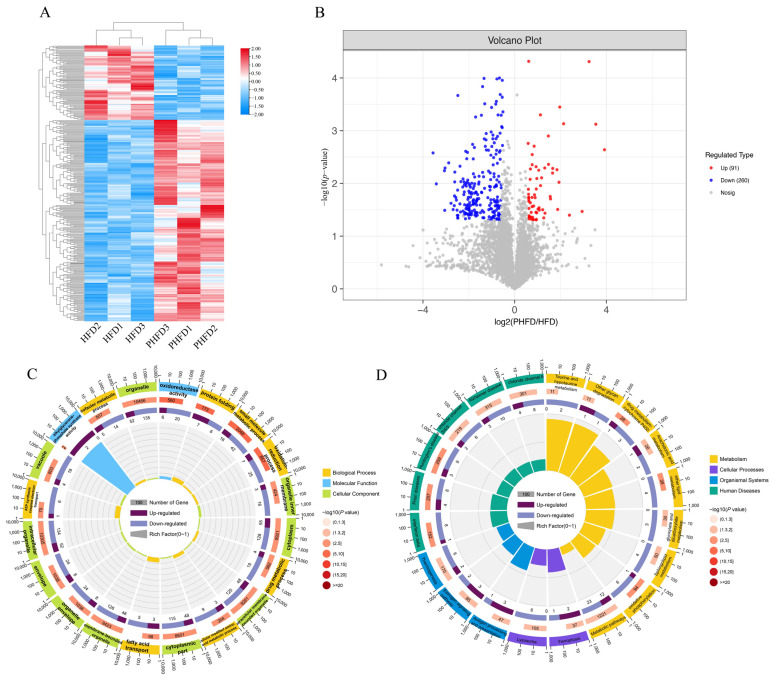
Statistical analysis of differentially expressed proteins (DEPs) between the high-fat diet group (HFD) and the probiotic supplementation group (PHFD) based on 4D-DIA proteomics (*n* = 6). (**A**) Heatmap of the expression of DEPs; (**B**) volcano plot of the expression of DEPs; functional enrichment of DEPs based on GO (**C**) and KEGG (**D**).

This study utilized 4D-DIA proteomics to collectively identify 31,213 peptide segments and 4774 proteins. Based on the fold change >1.5 or <0.67 and *p* < 0.05 criteria, a total of 351 DEPs were screened (Figure 3A), among which 91 were upregulated and 260 were downregulated (Figure 3B). GO enrichment analysis showed that these DEPs were mainly enriched in the organelles, intracellular organelles, and membrane-bounded organelles (Figure 3C). KEGG enrichment analysis showed that these DEPs were mainly enriched in the metabolism pathways (Figure 3D).

### 3.4. Identification of the DEPs Involved in Lipid Metabolism

**Figure 4:**
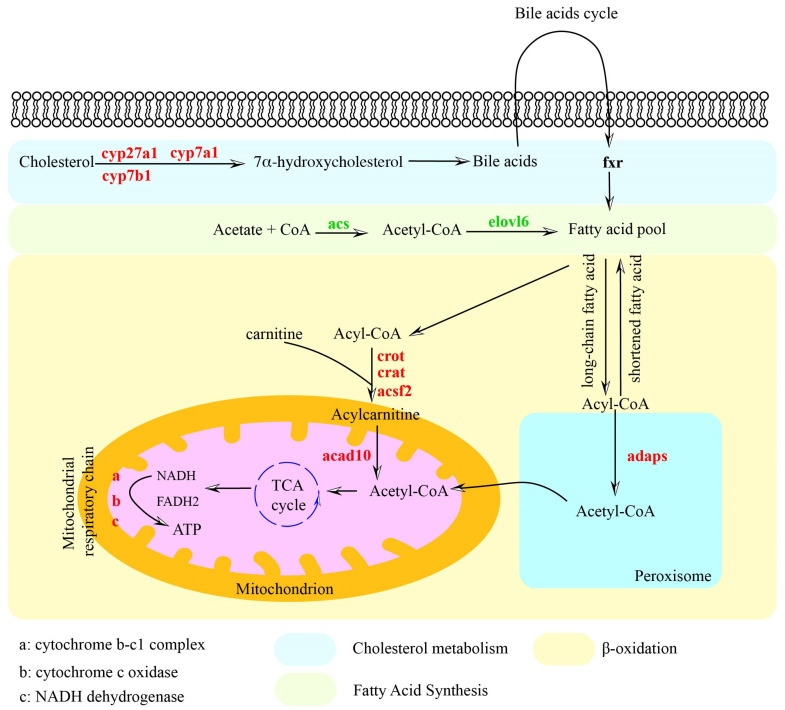
Probiotic-mediated impact pathways in the hepatic lipid metabolism in the *C. nasus* fed high-fat diets. Proteins indicated in red signify upregulation in the PHFD group, while those in green denote downregulation in the PHFD group. Metabolites (not detected) and organelles are represented in black.

Based on the potential functions of differential proteins, we identified 13 differential proteins involved in cholesterol metabolism, fatty acid biosynthesis, and β-oxidation (Table 1). The DEPs related to cholesterol metabolism and fatty acid β-oxidation were significantly upregulated, while those related to fatty acid biosynthesis were significantly downregulated. By reviewing the literature and the potential functions of differential proteins, we analyzed the potential relationship between probiotics and the modulation of lipid metabolism pathways in the liver of *C. nasu*s (Figure 4).

### 3.5. Identification of Immune-Related DEPs

Based on the potential functions of differential proteins, we screened out 12 differential proteins involved in immune regulation, including antioxidation, inflammatory response, and cellular autophagy (Table 2). Antioxidant-related DEPs were significantly upregulated. Among the inflammatory-related DEPs, plpp3 and sap were significantly upregulated, while lta4h and cpla2 were significantly downregulated. Cellular autophagy-related DEPs were significantly upregulated.

## 4. Discussion

Lipids serve as indispensable nutrients, supplying the requisite energy for the growth and developmental processes of aquatic organisms. Optimal levels of lipids have a dual effect on conserving proteins and promoting the absorption of other nutrients. The liver serves as the principal site for lipid metabolism, and fatty acid release is considered a major cause of obesity-related metabolic disorders [31]. The concentrations of TG, CHO, LDL-C, and HDL-C are used to assess lipid metabolism [32]. In this study, the supplementation of HFDs with probiotics led to a significant decrease in the levels of TG, CHO, and LDL-C in the *C. nasus* liver. Additionally, HFDs typically trigger interruptions of fat metabolism and excessive lipid accumulation, culminating in the onset of hepatic steatosis [33]. Adding probiotics to the HFDs significantly decreased the lipid vacuoles in the *C. nasus* liver, which indicated that adding probiotics to HFDs can effectively reduce liver fat accumulation and reduce fatty liver disease. Similar to our results, joint application of quercetin and hydroxytyrosol to HFDs reduced the TG, CHO, and NEFA levels in the liver, as well as the number of lipid droplets showing lipid deposition and reduced lipid toxicity in spotted seabass (*Lateolabrax maculatus*) [34]. Probiotics ameliorated excessive lipid accumulation induced by the HFDs in the *C. nasus* liver.

The fatty acid oxidation and de novo synthesis constitute pivotal metabolic routes that regulate lipid homeostasis, exerting significant influence on the onset and progression of hepatic steatosis [35]. Probiotics significantly upregulated the expression of the DEPs related to cholesterol metabolism, including cyp27a1, cyp7a1, and cyp7b1, which are key enzymes that metabolize cholesterol into bile acids [36]. Studies have indicated that probiotics enhance the hepatic transformation of cholesterol into bile acids, consequently decreasing cholesterol concentrations [19]. These results indicated that adding probiotics to HFDs promoted cholesterol metabolism, thereby decreasing the CHO level and increasing the bile acid level. Bile acids undergo enterohepatic circulation, being reabsorbed in the intestinal tract, re-entering the bloodstream, and subsequently returning to the liver [37]. The inclusion of bile acids in HFDs has been shown to markedly decrease lipid content within the hepatopancreas by regulating lipid catabolism [38]. Therefore, it was implied that cholesterol metabolism enhanced by probiotics improved bile acid synthesis to regulate lipid metabolism in *C. nasus* liver. The expression of DEPs related to fatty acid biosynthesis (acs and elovl6) was significantly downregulated after adding probiotics to the HFDs. Acetyl-coenzyme A synthetase is responsible for the synthesis of acetyl-coenzyme A, which is an essential substrate for de novo fatty acid synthesis [39]. ELOVL6 is a key enzyme involved in fatty acid elongation and synthesis [40]. The findings suggested that the addition of probiotics could inhibit fatty acid synthesis in the *C. nasus* liver, which was similar to the research on *M. amblycephala* [41]. Moreover, adding probiotics to the HFDs significantly upregulated the expression of crat, crot, acsf2, acad10, adaps, cytochrome b–c1 complex, cytochrome c oxidase, and NADH dehydrogenase, where crat, crot, and acsf2 participated in transporting long-chain fatty acids into mitochondria for beta-oxidation via carnitine metabolism [42]. ACAD10 is responsible for the oxidation of long-chain fatty acids, converting them into acetyl-CoA [43]. Subsequently, acetyl-CoA is channeled into the TCA cycle, where it generates NADH and FADH2, serving as crucial cofactors in the mitochondrial electron transport chain [44]. Peroxisomes, serving as another critical organelle for fatty acid β-oxidation, involve the participation of adaps in their intracellular fatty acid β-oxidation pathway [45]. These DEPs enhanced the fatty acid β-oxidation through the mitochondrial and peroxisomal pathways in the *C. nasus* liver. Consistent with our findings, the addition of resveratrol has been shown to suppress the fatty acid synthesis and stimulate β-oxidation in *M. amblycephala* and *L. maculatus* fed HFDs [46,47]. In short, adding probiotics to the HFDs mitigated hepatic lipid accumulation through promoting fatty acid β-oxidation and inhibited its biosynthesis in the *C. nasus* liver.

HFDs induced excessive ROS generation, consequently causing oxidative stress within biological systems [48]. Oxidative stress is primarily caused by a prolonged elevation of free fatty acid levels beyond the metabolic capacity [49]. Oxidative stress occurred in *Micropterus salmoides* and *M. amblycephala* fed HFDs [50,51]. Adding probiotics to the HFDs activated the CAT and SOD activities, and reduced the MDA level. In addition, it also significantly upregulated hsp70, gpx7, grx1, dnaja4, and pp2a. HSP70 was activated under the tissue damage and other stresses involved in the cell protection and repair processes [52]. DNAJA4 forms a complex with heat shock proteins (HSPs), enhancing the activity of HSPs and aiding in the correct recognition, binding, and folding of damaged proteins within the cell [53]. GPx7 catalyzes the reduction reaction of peroxides using the reducing agent glutathione [54]. Grx1 can protect cells from damage caused by oxidative stress, maintaining normal cellular functions [55]. PP2A participates in DNA repair, genomic stability, and immune responses [56]. These results indicated that probiotics enhanced the antioxidant activity and mitigated oxidative stress and liver damage induced by the HFDs in *C. nasus*. Similar to our results, bile acid supplementation to HFDs decreased the MDA levels and increased the mRNA expression of SOD and GSH-Px in *C. idella* [38] and *O. niloticus* [57]. The sustained progression of oxidative stress inevitably exacerbates inflammatory responses [58]. Adding probiotics significantly upregulated the expression of sap, while lta4h and cpla2 were significantly downregulated. LTA4H catalyzes the conversion of LTA-4 to LTB4, which is a potent chemoattractant leukotriene playing a crucial role in recruiting and activating inflammatory cells during the inflammation process [59]; cPLA2 hydrolyzes phosphatidylcholine to generate a series of inflammatory mediators, such as leukotriene B2 and LTB4 [60]; SAP can bind to IL-1β and IL-6, regulating their production and release [61]. Dietary choline improved hepatocyte function in *Acanthopagrus schlegelii* by inhibiting NF-κB activation, thereby alleviating inflammation induced by HFDs [6]. Adding L-carnitine to HFDs was found to downregulate TNF-α and IL-1β in the hepatic tissue of zebrafish (*Danio rerio*) [62]. Autophagy serves as the foundation for regulating hepatic lipid metabolism [63]. In addition, adding probiotics to the HFDs significantly downregulated wdfy1 and tmem11. WDFY1 regulates the association of organelles and membranes for the autophagosome formation and the membrane extension [64]. TMEM11 participates in the regulation of mitochondrial autophagy by interacting with PINK1 and Parkin [65]. These results indicated that adding probiotics to the HFDs inhibited autophagy in the *C. nasus* liver. HFDs diminish hepatic autophagy, leading to increased lipid accumulation within the hepatic tissue. Long-term HFDs activated hepatic autophagy via regulating the inositol-requiring enzyme-1 (IRE1) and AMPK signaling pathway [9,66]. Contrary to our results, dietary selenium (Se) alleviates hepatic and pancreatic lipid accumulation induced by HFDs through the activation of lipophagy in *C. idella* [67]. Due to the lack of currently published research on farmed fish to investigate the correlation between autophagy and hepatic lipidosis induction, we inferred that different treatment conditions may lead to cells choosing different types of autophagic pathways, such as microautophagy, macroautophagy, mitophagy, or lipophagy. In brief, adding probiotics to the HFDs enhanced the antioxidant activity and inhibited inflammation as well as autophagy in *C. nasus*.

## 5. Conclusions

In short, adding probiotics to the HFDs decreased the lipid vacuoles to reduce lipid accumulation in the *C. nasus* liver. Proteomics analysis demonstrated that adding probiotics to the HFDs promoted cholesterol metabolism as well as fatty acid β-oxidation and inhibited fatty acid biosynthesis in the *C. nasus* liver to reduce lipid accumulation. Moreover, adding probiotics to the HFDs increased the CAT and SOD levels and depressed the MDA level, as well as the ALT and AST activities to enhance the antioxidant activity and inhibit liver damage. Proteomics analysis showed that adding probiotics to the HFDs enhanced the antioxidant activity and inhibited inflammation as well as autophagy in *C. nasus*. Our studies enhance the understanding of the nutritional and physiological functions of probiotic supplementation to high-fat diets in fish, laying the foundation for further research on the metabolic regulation mechanism of probiotics in fish and providing a basis for the effective utilization of high-fat diets in *C. nasus*.

## Figures and Tables

**Table 1:** DEPs related to lipid metabolism in the *C. nasus* liver in the high-fat diet group (HFD) and the probiotic supplementation group (PHFD).

Category	Protein Name	log_2_ FC (PHFD vs. HFD)	*p*-Value
Cholesterol metabolism	cyp27a1	1.03799	0.04690
	cyp7a1	1.61418	0.01628
	cyp7b1	0.86079	0.04051
Fatty acid synthesis	acs	−0.82762	0.02826
	elovl6	−3.54031	0.00075
β-oxidation	crot	0.89933	0.04389
	crat	0.77788	0.00496
	acsf2	0.60992	0.02569
	acad10	3.01985	0.04275
	adaps	0.82102	0.04862
	cytochrome b-c1 complex	0.58690	0.01976
	cytochrome c oxidase	2.77624	0.02383
	NADH dehydrogenase	0.75456	0.00415

Note: cyp27a1: cytochrome P450 27A1, cyp7a1: cytochrome P450 7A1, cyp7b1: cytochrome P450 7B1, acs: acetyl-coenzyme A synthetase, cytoplasmic, elovl6: elongation of very long-chain fatty acids protein 6, crat: carnitine O-acetyltransferase, crot: carnitine O-octanoyltransferase, acsf2: medium-chain acyl-CoA ligase ACSF2, mitochondrial, acad10: acyl-CoA dehydrogenase family member 10, adaps: alkyldihydroxyacetonephosphate synthase, peroxisomal.

**Table 2:** DEPs related to immunity in the *C. nasus* liver in the high-fat diet group (HFD) and the probiotic supplementation group (PHFD).

Category	Protein Name	log_2_ FC (PHFD vs. HFD)	*p*-Value
Antioxidant	hsp70	3.24694	0.00005
	gpx7	1.38751	0.04474
	grx1	0.59686	0.04087
	dnaja4	1.96795	0.00036
	pp2a	1.87770	0.03120
Inflammation	lta4h	−0.61626	0.02037
	cpla2	−0.81500	0.02278
	sap	0.98291	0.04948
Autophagy	wdfy1	−0.78409	0.02599
	tmem11	−0.64195	0.00794

Note: hsp70: heat shock 70 kDa protein, gpx7: glutathione peroxidase 7, grx1: glutaredoxin-1, dnaja4: dnaJ homolog subfamily A member 4, pp2a: serine/threonine-protein phosphatase alpha-2, lta4h: leukotriene A-4 hydrolase, cpla2: cytosolic phospholipase A2, sap: serum amyloid P-component, wdfy1: WD repeat and FYVE domain-containing protein 1, tmem11: transmembrane protein 11, mitochondrial.

## Data Availability

The proteomics data were submitted to the iProX database (project ID: IPX0008644000).

## Supplementary Materials

The following supporting information can be downloaded at: https://www.mdpi.com/article/10.3390/biology14040381/s1, Table S1: Ingredient composition and analysis of the experimental diets (%).

## Abbreviations

The following abbreviations are used in this manuscript:

TG: triglycerides; CHO: cholesterol; LDL-C: low-density lipoprotein cholesterol; AST: aspartate aminotransferase; ALT: alanine aminotransferase; MDA: malondialdehyde; CAT: catalase; SOD: superoxide dismutase; scd: stearoyl-CoA desaturase; srebp1: sterol regulatory element-binding protein 1; hsl: hormone-sensitive lipase; pparα: peroxisome proliferator-activated receptors; cpt1: carnitine palmitoyltransferase 1; HDL-C: high-density lipoprotein cholesterol; GPx: glutathione peroxidase; TNF-α: tumor necrosis factor alpha; IL-1β: interleukin 1 beta; ROS: reactive oxygen species; cyp27a1: cytochrome P450 27A1; cyp7a1: cytochrome P450 7A1; cyp7b1: cytochrome P450 7B1; acs: acetyl-coenzyme A synthetase, cytoplasmic; elovl6: elongation of very long-chain fatty acids protein 6; crat: carnitine O-acetyltransferase; crot: carnitine O-octanoyltransferase; acsf2: medium-chain acyl-CoA ligase ACSF2, mitochondrial; acad10: acyl-CoA dehydrogenase family member 10; adaps: alkyldihydroxyacetonephosphate synthase, peroxisomal; cbc1c: cytochrome b–c1 complex; cco: cytochrome c oxidase; NADHd: NADH dehydrogenase; hsp70: heat shock 70 kDa protein; gpx7: glutathione peroxidase 7; grx1: glutaredoxin-1; dnaja4: dnaJ homolog subfamily A member 4; pp2a: serine/threonine-protein phosphatase alpha-2; lta4h: leukotriene A-4 hydrolase; cpla2: cytosolic phospholipase A2; sap: serum amyloid P-component; wdfy1: WD repeat and FYVE domain-containing protein 1; tmem11: transmembrane protein 11, mitochondrial.
