# Supplementary material for: Probiotic Supplementation Improves Lipid Metabolism Disorders and Immune Suppression Induced by High-Fat Diets in Coilia nasus Liver
Source: Biology (Basel). 2025 Apr 7;14(4):381. doi: 10.3390/biology14040381 (PMC12024547; doi:10.3390/biology14040381)
Supplement: Supplementary file 1 [file biology-14-00381-s001.zip › biology-3559549-supplementary.pdf]

Table S1 Ingredients composition and analysis of the experimental diets (%)

Ingredients	Percent
Fish meal	48
Soybean meal	22
Wheat flour	11.5
Fish oil	4.5
Soybean oil	2
Soybean lecithin	2
Vitamin and Mineral mix	4
Dicacium phosphate	1
Choline chloride	1
Microcrystalline cellulose	4
Moisture	8.65 ± 0.08
Crude protein	47.18 ± 2.04
Crude fat	14.58 ± 0.91
Ash	13.39 ± 0.18
